# Using machine learning models to predict post-revascularization thrombosis in PAD

**DOI:** 10.3389/frai.2025.1540503

**Published:** 2025-05-07

**Authors:** Samir Ghandour, Adriana A. Rodriguez Alvarez, Isabella F. Cieri, Shiv Patel, Mounika Boya, Rahul Chaudhary, Anna Poucey, Anahita Dua

**Affiliations:** ^1^Division of Vascular and Endovascular Surgery, Massachusetts General Hospital, Boston, MA, United States; ^2^Division of Cardiology, Heart and Vascular Institute, University of Pittsburgh Medical Center, Pittsburgh, PA, United States; ^3^Division of Cardiology, Veterans Affairs Pittsburgh Health System, Pittsburgh, PA, United States; ^4^AI-HEART Lab, Pittsburgh, PA, United States; ^5^Division of Vascular Surgery, Imperial College London, London, United Kingdom

**Keywords:** thromboelastography with platelet mapping, revascularization, prognosis, machine learning, thrombosis

## Abstract

**Background:**

Graft/ stent thrombosis after lower extremity revascularization (LER) is a serious complication in patients with peripheral arterial disease (PAD), often leading to amputation. Thus, predicting arterial thrombotic events (ATE) within 1 year is crucial. Given the high rates of thrombosis post-revascularization, this study aimed to develop a machine learning model (MLM) incorporating viscoelastic testing and patient-specific variables to predict ATE following LER.

**Methods:**

We prospectively enrolled PAD patients undergoing LER from 2020 to 2024, collecting demographic, clinical, and intervention-related data alongside perioperative thromboelastography with platelet mapping (TEG-PM) values over 12 months post-revascularization. Univariate analysis identified predictors from 52 candidate variables. Multiple MLMs, including logistic regression, XGBoost, and decision tree algorithms, were developed and evaluated using a 70–30 train-test split and five-fold cross-validation. The Synthetic Minority Oversampling Technique (SMOTE) was employed to address the class imbalance between the primary outcomes (ATE vs. no ATE). Model performance was assessed by area under the curve (AUC), accuracy, sensitivity, specificity, negative predictive value, and positive predictive value.

**Results:**

Of the 308 patients analyzed, 66% were male, 84% were White, and 18.3% experienced an ATE during the one-year post-revascularization follow-up period. The logistic regression MLM demonstrated the best combined descriptive and calibration performance, especially when TEG-PM parameters were used in combination with patient-specific baseline characteristics, with an AUC of 0.76, classification accuracy of 70%, sensitivity of 68%, and specificity of 71%.

**Conclusion:**

Combining patient-specific characteristics with TEG-PM values in MLMs can effectively predict ATE following LER in PAD patients, enhancing high-risk patient identification and enabling tailored thromboprophylaxis.

## Introduction

Peripheral arterial disease (PAD) affects over 200 million people globally, constituting a significant burden on healthcare and patient quality of life (Song et al., 2019; Shu and Santulli, 2018; Sorber et al., 2021). Severe PAD management involves lower extremity graft or stent revascularization procedures (Kinlay, 2016; Hicks et al., 2017). Despite advances, bypass graft and/or stent thrombosis within the first year remains common, occurring between 5 and 20% of cases (Majumdar et al., 2023). Arterial thrombotic events (ATE) remain a leading cause of limb loss, morbidity, and mortality following revascularization, with up to an estimated 50% mortality rate within 1 year of amputation (Beeson et al., 2023; Andraska et al., 2022). The risk of ATE post-revascularization is influenced by a complex interplay of patient-specific factors, including genetics and comorbid conditions such as diabetes mellitus, hypertension, renal insufficiency, and smoking status, which all influence the intrinsic coagulation status of a patient (Brooke et al., 2014; O'Hare et al., 2004). Traditional risk assessment tools, while useful, often fall short of accurately and reliably predicting these events, necessitating a more individualized approach. Specifically, the role of patient hypercoagulability and its management remains poorly characterized. Despite significant research into the molecular biology of cardiovascular disease in general, there is limited evidence supporting the use of multimodal antithrombotic therapy specifically for PAD to alleviate post-revascularization thrombosis or stenosis of the index arterial lesion. Notably, studies such as VOYAGER PAD have explored strategies to improve thromboprophylaxis in PAD patients. However, VOYAGER intervention (aspirin + rivaroxaban) did not significantly decrease major amputation rates as an individual outcome and was associated with an increased risk of major bleeding, limiting its suitability as a universal approach (Berkowitz et al., 2022). Additionally, most of the trials were derived from subgroup analysis of patients with coronary and cerebrovascular disease (Sorber et al., 2021; Berkowitz et al., 2022; Dorweiler et al., 2003; Kohler et al., 1984; Steffel et al., 2020).

Many factors, such as antiplatelet agent resistance, uremia, and glycemic control, may increase thrombosis risk. However, they are not adequately accounted for in standard hypercoagulability tests (prothrombin time, international normalized ratio, and activated partial thromboplastin time). These tests measure individual steps of the coagulation cascade in a non-physiologic setting and can poorly reflect the propensity for *in vivo* coagulation. These metrics do not measure the effectiveness of commonly used antithrombotic agents in PAD. Thromboelastography with platelet mapping (TEG-PM), an advanced hemostatic testing method, offers a comprehensive overview of a patient’s coagulation status (Majumdar et al., 2023). TEG-PM evaluates the kinetics of clot formation, strength, and lysis, providing insights far beyond what is possible with conventional coagulation tests. The predictive value of TEG-PM in various clinical settings, including perioperative management and trauma, has been increasingly recognized (Majumdar et al., 2022; Bugaev et al., 2020). However, its utility in the specific context of lower extremity revascularization (LER) and ATE risk prediction following intervention remains underexplored but promising, given the potential of escalated antithrombotic management for those at elevated risk of ATE (Majumdar et al., 2023). One current drawback of utilizing TEG-PM is that even though a significant amount of patient coagulation data can be obtained, there is a limited understanding of the significance of its parameters and which cutoff values are thought to denote elevated risk ATE incidence following arterial LER.

Machine learning models (MLM) are increasingly utilized in the medical field for outcome event prediction and clinical decision support. A significant strength of many MLMs is their ability to infer complex interactions between multiple factors without pre-specification. This property of MLMs is especially impactful in the setting of multiple predictors of previously uncharacterized significance and their potential complex interaction leading to a specific outcome. Currently, there are no models that utilize patient-specific baseline factors and quantitative coagulability data to predict ATE following lower extremity revascularization. This study aims to develop an MLM using relevant baseline patient-specific variables (clinical, demographic, and intervention-related) and TEG-PM values that could accurately predict ATE within 1 year of LER. Moreover, this study aims to evaluate the efficacy of MLMs incorporating TEG-PM data compared to those based solely on traditional risk factors, mimicking clinician assessment. We hypothesize that the addition of TEG-PM values for this approach utilizing prospectively collected patient data enhances the predictive capacity for ATE, potentially guiding tailored interventions to mitigate the risk of ATE post-revascularization in high-risk patients with PAD.

## Methods

### Study design and population

This prospective longitudinal study enrolled consecutive symptomatic PAD patients undergoing LER at a single tertiary medical center in the United States between December 2020 and March 2024. This study was approved by the Institutional Review Board (Mass General Brigham IRB#2022P001918). Written or electronic consent was obtained from all study participants, or their legally authorized representative, before enrollment.

Inclusion criteria for enrollment include patients above 18 years who underwent LER, history of atherosclerotic disease, and evidence of PAD on imaging or ankle-brachial-index (ABI) assessment upon presentation with symptoms including claudication, pain, distal ulceration, or gangrene. Patients with the inability to undergo serial blood draws, provide informed consent, are pregnant, or did not have a successful revascularization were excluded.

Successful revascularization was achieved when adequate blood flow was restored to an ischemic area by bypassing and treating the atherosclerotic blockage using methods such as angioplasty, stenting, or bypass surgery. Patients underwent revascularization based on assessments by a board-certified vascular surgeon.

### Blood sample collection

Blood samples were collected preoperatively within 24 h of intervention (baseline), and at 1 month, 3 months, 6 months, and 12 months postoperatively for TEG-PM analysis. Blood collection was performed via a peripheral stick, using a 4.0-mL sodium heparin and citrated vacutainer, followed by 10–30 min of incubation and analysis within 2–4 h per the manufacturer’s instructions.

### TEG-PM analysis

Whole blood samples were tested with the TEG6 S Hemostasis Analyzer (Haemonetics Corp, Boston MA). Citrated multichannel cartridges without lysis, measuring time to clot formation (K-time), cloth strengthening (K-time and *α*-angle), and maximum amplitude (MA) were utilized. Platelet Mapping cartridges were assayed with heparinized blood to quantify platelet function in response to ADP agonists. Platelet function quantification with TEG-PM is based on the principle that the difference between the MA and the contribution of fibrinogen to clot strength may be considered an index of platelet contribution to clot strength. The Platelet Mapping cartridge consists of dried-in-place reagents to calculate the MA in various scenarios: a standard kaolin-activity thromboelastography, which is considered “best platelet reactivity”; a pure fibrin clot by adding reptilase, which directly converts fibrinogen to fibrin and corresponds to 0% platelet contribution; and an ADP-activated clot to detect platelet reactivity in the presence of aspirin or P2Y12 inhibition. Thus, platelet reactivity (percentage) is calculated as follows: 100 × MAADP/(MAKaolin–MAFibrin). If an ATE occurs, the nearest TEG-PM sample analysis performed prior to the onset of the event was used for that case to train the prediction model.

TEG-PM evaluations, along with routine clinical lab evaluations, were performed for all participants at several time points. For participants who did not sustain an ATE, the baseline preoperative and most recent values were associated with participants’ records. For patients who experienced an ATE, the baseline preoperative and temporally closest TEG-PM evaluation to the time of ATE occurrence was associated with the participants’ records.

### Candidate predictive variables

Baseline demographic, clinical, and intervention-related patient data were collected. Baseline demographic information includes age, sex, race, smoking status, and body mass index (BMI). Baseline clinical data including systolic blood pressure, Rutherford score, ankle-brachial index of the affected extremity, lesion site, intervention type, antiplatelet therapy, anticoagulant therapy, statin therapy, complete blood count values, platelet time (PT), International Normalized Ratio (INR), and partial thromboplastin time (PTT) were recorded.

Comorbid conditions such as diabetes, hypertension, hyperlipidemia, coronary artery disease, and chronic kidney disease were recorded as categorical variables. Patient clinical history of myocardial infarction, pulmonary embolism, deep venous thrombosis, malignancy, previously occluded bypass graft or stent, and stroke were recorded as well. Target lesion revascularization intervention characteristics were assessed and tracked. The intervention type was categorized as open surgery; endovascular; or hybrid (both open and endovascular interventions). The target arterial lesion was categorized as either aortoiliac, suprapopliteal, or infrapopliteal.

### Primary outcome

The primary outcome of interest, ATE within 1 year following revascularization, was categorized as a binary variable to generate a binary classification predictive model. ATE was defined as a composite outcome of graft/stent thrombosis on radiographic imaging, index lesion occlusion requiring re-intervention to maintain or re-establish arterial flow or major limb amputation due to extensive unsalvageable index arterial lesion thrombosis during post-revascularization follow-up.

### Final dataset and preprocessing

Fifty-two candidate predictor variables were analyzed for inclusion in the final predictive model: 20 clinical, 8 intervention-specific, and 24 TEG-PM parameters. All candidate tables are listed in Tables 1–3, and Supplementary Table S1. Variables were classified as categorical or continuous, accordingly. Label encoding was used to convert categorical variables into numerical values for model input. Variables with more than 50% missing values were excluded, and the remaining missing values were imputed with calculated mean values separately within the ATE and non-ATE groups to preserve potential differences in data distribution and to avoid homogenizing values across outcome groups.

**Table 1:** Baseline demographic, clinical, and historical characteristics of enrolled participants.

Variable	No ATE group (*n* = 252)	ATE group (*n* = 56)	Total (*n* = 308)	*p*-value
Age (years)	69.7 (10.4)	68.0 (11.1)	69.4 (10.6)	0.31
Sex (female)	87 (34.5)	18 (32.1)	105 (34.1)	0.85
Race (White)	46 (82.1)	223 (88.5)	269 (87.3)	0.0.29
BMI (kg/m^2^)	27.1 (5.6)	27.4 (6.5)	27.2 (5.7)	0.77
Diabetes	132 (52.4)	36 (64.3)	168 (54.5)	0.14
Hypertension	227 (90.1)	50 (89.3)	277 (89.9)	1.0
Chronic kidney disease				0.25
Stage 0	82 (32.5)	26 (46.4)	108 (35.1)	–
Stage 1	24 (9.5)	2 (3.6)	26 (8.4)	–
Stage 2	69 (27.4)	12 (21.4)	81 (26.3)	–
Stage 3	57 (22.6)	13 (23.2)	70 (22.7)	–
Stage 4	7 (2.8)	0 (0)	7 (2.3)	–
Stage 5	13 (5.2)	3 (5.4)	16 (5.2)	–
Coronary artery disease	136 (54.0)	30 (53.6)	166 (53.9)	1.0
History of MI	66 (26.2)	17 (30.4)	83 (26.9)	0.64
Functional impairment	115 (45.6)	33 (58.9)	148 (48.1)	0.10
Clotting disorder	9 (3.6)	1 (1.8)	10 (3.2)	0.66
Active malignancy	23 (9.1)	3 (5.4)	26 (8.4)	0.44
History of malignancy	60 (23.8)	12 (21.4)	72 (23.4)	0.84
History of DVT	31 (12.3)	10 (17.9)	41 (13.3)	0.37
History of stroke	40 (15.9)	12 (21.4)	52 (16.9)	0.42
History of pulmonary embolism	11 (4.4)	2 (3.6)	13 (4.2)	1.0
Previous revascularization of index limb	105 (41.7)	36 (64.3)	141 (45.8)	0.003
History of stent occlusion	35 (13.9)	14 (25.0)	49 (15.9)	0.03
Rutherford score	3.8 (1.4)	4.3 (1.2)	3.9 (1.4)	0.004
ABI of affected lower extremity	1.2 (1.3)	0.8 (0.3)	1.1 (0.6)	0.38
Artery affected – Infrapopliteal	129 (51.2)	42 (75.0)	171 (55.5)	0.002
Artery affected – Suprapopliteal	196 (77.8)	42 (75.0)	238 (77.3)	0.79
Artery affected – Aortoiliac	50 (19.8)	1 (1.8)	51 (16.6)	0.002
Endovascular revascularization	138 (54.8)	32 (57.1)	170 (55.2)	0.86
Open revascularization	69 (27.4)	14 (25.0)	83 (26.9)	0.84
Combined revascularization	44 (17.5)	10 (17.9)	54 (17.5)	1.0
Active smoker	12 (4.8)	0 (0)	12 (3.9)	0.242
Aspirin therapy	181 (80.8)	44 (74.6)	225 (79.5)	0.38
Clopidogrel therapy	117 (52.2)	35 (59.3)	152 (53.7)	0.41
Atorvastatin therapy	215 (76.0)	161 (71.9)	54 (91.5)	0.003
Baseline systolic blood pressure	107.4 (54.4)	130.9 (24.9)	112.3 (50.6)	<0.001

Data are presented as *n* (%) or mean ± standard deviation (SD). BMI, body mass index; MI, myocardial infarction; DVT, deep vein thrombosis; ABI, ankle-brachial index.

**Table 2:** TEG-PM parameter values for all participants.

TEG-PM parameter	No ATE group (*n* = 252)	ATE group (*n* = 56)	Total (*n* = 308)	*p*-value
Reaction time (R) in min	7.7 (3.5)	8.7 (3.9)	7.9 (3.6)	0.09
Lysis at 30 min (LY30) in %	0.8 (1.7)	0.5 (0.7)	0.7 (1.6)	0.13
CRT Max amplitude (MA) in mm	64.5 (7.0)	65.2 (6.3)	64.6 (6.9)	0.41
CFF Max Amplitude (MA) in mm	28.0 (10.6)	28.9 (11.2)	28.2 (10.7)	0.57
HKH MA (mm)	60.6 (9.7)	59.7 (10.2)	60.4 (9.8)	0.53
ActF MA (mm)	15.3 (7.9)	15.4 (8.1)	15.4 (7.9)	0.98
ADP MA (mm)	44.0 (18.0)	42.3 (17.6)	43.6 (17.9)	0.52
AA MA (mm)	32.8 (20.9)	32.5 (18.2)	32.7 (20.3)	0.92
CK R (min)	7.6 (3.2)	8.4 (4.0)	7.8 (3.4)	0.15
CK K (min)	1.8 (1.3)	1.9 (1.3)	1.8 (1.3)	0.67
CK angle (deg)	69.6 (10.2)	68.4 (10.4)	69.3 (10.2)	0.45
CK MA (mm)	62.2 (7.8)	61.2 (8.6)	62.0 (8.0)	0.43
CRT MA (mm)	63.7 (8.7)	64.8 (6.3)	63.9 (8.3)	0.26
CKH R (min)	7.2 (3.4)	7.1 (1.9)	7.2 (3.1)	0.64
CFF MA (mm)	28.0 (11.1)	28.5 (11.5)	28.1 (11.1)	0.76
CFF FLEV (mg/dL)	484.6 (164.0)	496.3 (182.6)	487.0 (167.8)	0.66

Data are presented as mean ± standard deviation (SD). AA Aggregation, Percentage of platelet aggregation induced by arachidonic acid; AA Inhibition, Percentage of platelet inhibition in response to arachidonic acid, often used to monitor COX inhibitors like aspirin; AA MA, Arachidonic Acid Maximum Amplitude, assesses platelet function influenced by arachidonic acid, related to cyclooxygenase (COX) activity; ActF MA, Activated Functional Maximum Amplitude, represents the overall clot strength in functional assays; ADP Aggregation, Percentage of platelet aggregation induced by ADP stimulation; ADP Inhibition, Percentage of platelet inhibition in response to ADP, often used to monitor antiplatelet therapy; ADP MA, Adenosine Diphosphate Maximum Amplitude, measures platelet contribution to clot strength mediated by ADP receptors; CFF FLEV, Coagulation Factor Fibrinogen Functional Level, a quantitative measure of functional fibrinogen in the blood; CFF MA, Coagulation Factor Fibrinogen Maximum Amplitude, measures the clot strength specifically attributed to fibrinogen; CK Angle, Citrated Kaolin Angle, the rate of clot formation, measured as the angle of the TEG curve; CK K, Citrated Kaolin Clot Formation Time, the time for the clot to reach a certain strength; CK MA, Citrated Kaolin Maximum Amplitude, the maximum clot strength; CK R, Citrated Kaolin Reaction Time, time to initial clot formation; CKH R, Citrated Kaolin Heparin Reaction Time; CRT MA, Citrated Rapid TEG Maximum Amplitude, maximum clot strength assessed with the rapid TEG protocol; HKH MA, Heparin Kaolin High Maximum Amplitude, assesses clot strength in the presence of heparin and kaolin as activators.

**Table 3:** The comparative performance metrics of each generated predictive model using the three prespecified datasets (baseline only, TEG-PM only, and baseline and TEG-PM combined).

Model	Accuracy	Sensitivity	Specificity	PPV	NPV	F1 Score	AUC
XGBOOST (baseline dataset)	67	34	74	24	82	0.25	0.52
XGBOOST (TEG-PM dataset)	57	64	55	29	86	0.39	0.66
XGBOOST (TEG-PM and baseline dataset)	65	68	55	30	85	0.41	0.68
Decision trees (baseline dataset)	66	38	72	22	84	0.26	0.50
Decision trees (TEG-PM dataset)	56	63	54	26	86	0.37	0.64
Decision trees (TEG-PM and baseline dataset)	54	75	49	28	88	0.40	0.63
Logistic regression (baseline dataset)	57	45	60	20	83	0.27	0.51
Logistic regression (TEG-PM dataset)	61	64	61	31	87	0.41	0.68
Logistic regression (TEG-PM and baseline dataset)	70	68	71	38	89	0.48	0.76

Data are presented as % and mean. PPV, positive predictive value; NPV, negative predictive value; AUC, area under the receiver operating characteristic (ROC) curve; TEG-PM, Thromboelastography with Platelet Mapping.

Univariate analysis of candidate predictor variables was done with respect to thrombotic events to identify significantly associated risk factors for selection and inclusion in model training, with practical clinical relevance taken into consideration in the selection process as well. Correlation and *p*-values were used for feature selection following model inclusion, and the variables with the highest correlation metrics were chosen from the initial 52 variables. A Chi-square Test was used for categorical variables. For continuous variables, a *t*-test or ANOVA test was used accordingly. A *p* < 0.1 was considered significant.

Three dataset modules were created for MLM training and testing, particularly selecting features that were significantly associated with ATE incidence. The first dataset included 16 selected baseline patient-specific variables (features) that are traditionally considered cardiovascular risk factors for thrombosis, mimicking clinical assessment. The second dataset included 13 selected TEG- PM variables clinical lab evaluations (features) that correlated significantly with the incidence of ATE. The third dataset module included 22 combined baseline and TEG-PM variables (features). For each MLM, each dataset was used separately for model training and testing, and the predictive performance of each MLM using the datasets was compared.

### Model fine-tuning and development

Multiple MLMs were developed with both feature importance and predictive performance compared. These models included Extreme Gradient Boosting (XGBoost), Logistic Regression (LR), and Decision Tree Algorithm. Given the predicted outcome of ATE happens in only a small minority of patients, resulting in an unbalanced dataset, there was potential for model bias toward predicting the non-event outcome (no ATE). Secondary analysis was performed utilizing the Synthetic Minority Oversampling Technique (SMOTE), which computationally generates additional events (ATE outcomes) for MLM training purposes to attempt to offset the imbalanced nature of the initial dataset. This enhanced the representation of ATE during model training to improve model generalizability and predictive performance.

A random 70–30 train-test dataset split logic was used to develop and evaluate the performance of the predictive MLMs. Since the datasets are relatively small, the smaller testing split may potentially lead to an unreliable performance estimate. To address this limitation and reduce overfitting, we employed five-fold cross-validation with a performance metric of receiver operating characteristic (ROC). This technique involves splitting the complete dataset into 5 folds and then iteratively training and testing the model on each fold, after which we averaged the performance metrics across these iterations This process provides a more reliable estimate of the model’s performance on unseen data by reducing the influence of specific data patterns that might not generalize well. Hyper-parameter tuning was conducted using a systematic grid search approach to work through multiple combinations of parameter tunes, cross-validating as it went to determine which parameters performed best. Modified hyper-parameter included learning rate, number of trees, depth of trees, and regularization terms.

### Model comparisons

After MLM generation, the performance of the different models was compared across accuracy, precision (PPV), recall (sensitivity), specificity, F1 score, and ROC AUC. Precision-recall curves were generated for each model to assess their performance in handling class (negative and positive) imbalances. For each model generated, optimized predictive cut-off values were determined using the PR-AUC curves accordingly.

### Feature importance and cut point analysis

Individual variable predictive importance was determined using permutation feature importance calculations in the training dataset. Permutation feature importance is determined after the final model is fitted to the training dataset and the training set predictive performance has been determined (ROC in these models). Each predictor in the training dataset is then randomly altered, and a new predictive performance (ROC) is assessed in this permuted training data. The feature importance is determined by the difference in ROC between the original model and the permutated model. Permutation feature importance is intuitive to interpret and independent of model type, allowing covariate importance comparisons between different models. Determining predictor importance by permutation methods has several drawbacks: (1) the determined importance scores are a relative measure of predictive power for each feature, and score comparison between predictive models from separate datasets is not meaningful; (2) high permutation feature importance does not necessarily indicate statistical inference or delineate the nature of the relationship between the predictor and the outcome (linear, quadratic, etc.); and (3) permutation feature importance can perform poorly in the setting of covariate collinearity, as only one predictor is altered for each permutation and the model may still perform well if a collinear variable remains unaltered.

## Results

A total of 308 patients were enrolled in the study. Their mean age was 69.4 (±10.6) years, 66% were male, and 84% were white. Among the participants prospectively enrolled, 252 (81.8%) did not experience an ATE (negative controls), and 56 (18.2%) experienced an ATE (positive cases) during the one-year follow-up period post-revascularization as shown in Table 1. There were no statistically significant differences in TEG parameters (Table 2) or laboratory values (Supplementary Table S1) between the ATE and non-ATE groups.

**Figure 1:**
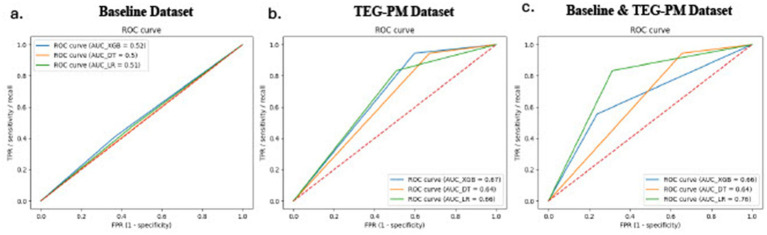
Receiver operating characteristic curves comparing the three predictive models across the three dataset. The receiver operating characteristic curves of the XGBOOST (blue), logistic regression (green), and decision trees (orange) models when trained using the baseline only **(a)**, TEG-PM only **(b)**, and combined baseline and TEG-PM datasets **(c)**.

**Figure 2:**
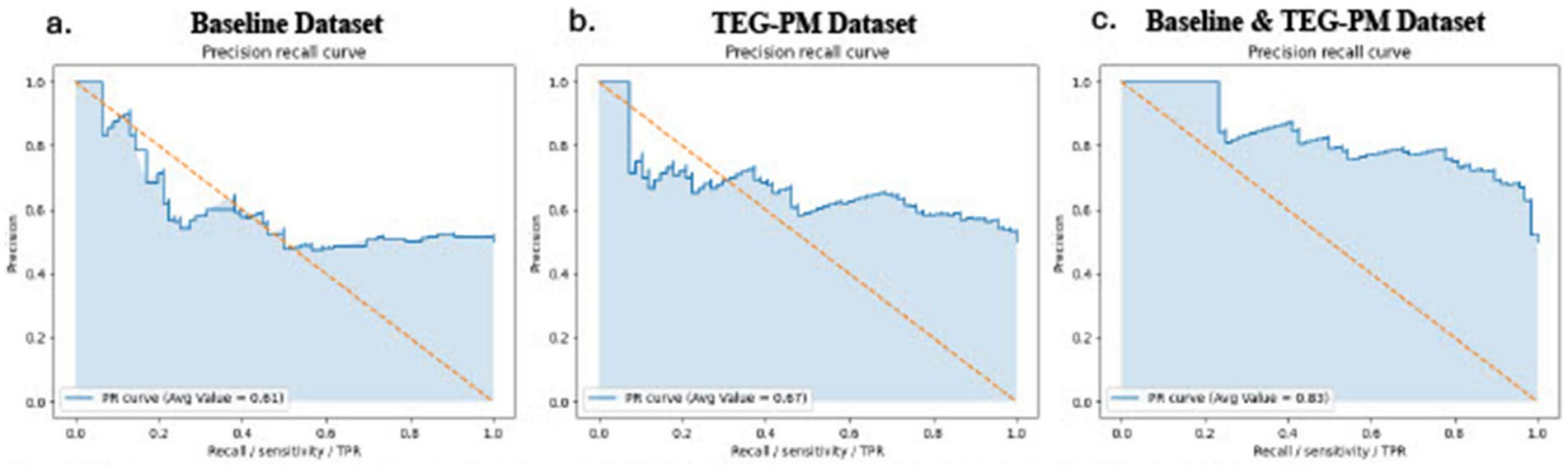
Precision-recall curves for the logistic regression model across the three datasets. The precision-recall curves for the logistic regression model, which was the best-performing prediction model, are shown for different datasets. The baseline dataset alone had an average precision-recall value of 0.61 **(a)**, the TEG-PM dataset alone had an average value of 0.67 **(b)**, and the combination of both baseline and TEG-PM datasets yielded the highest average value of 0.83 **(c)**.

The MLM training set contained data from 216 random patients (70%), and the MLM testing set contained 92 random patients (30%). Among all three developed MLMs (XGBOOST, Decision Trees, and LR), the LR model showed the highest predictive performance by both discrimination metrics (AUC ROC) and calibration analysis. All three models performed poorly when trained using only patient-specific baseline variables but performed better when trained using TEG-PM variables. The LR model showed the best prediction performance compared to the XGBoost and Decision Trees models, especially when traditional baseline variables are combined with TEG-PM parameters collected periodically over the course of follow-up (Figures 1, 2). However, when the LR MLM was trained only using baseline characteristics it performed relatively poorly with an AUC of 0.51. On the other hand, when the LR MLM was trained using only the most relevant TEG-PM parameters, it showed better performance with an AUC of 0.68. MLM predictive performance for all generated models is listed in Table 3.

## Discussion

Currently, there are no reliable scoring systems utilized to anticipate the risk of lower extremity ATEs for patients with PAD post-revascularization. Treating surgeons primarily rely on their clinical experience and literature knowledge describing risk factors associated with cardiac and intracranial arterial thrombosis to anticipate high-risk individuals. This has been shown to be ineffective; even though patients with PAD share similar cardiovascular risk factors for thrombosis, this patient population is markedly heterogeneous and presents with highly variable profiles that may impact thrombotic outcomes. Other coagulative conditions, such as deep venous thrombosis (DVT) have scoring systems for predicting its risk (Caprini and Wells scores) (Modi et al., 2016; Grant et al., 2016). Therefore, the ability to anticipate such catastrophic complications objectively and reliably is paramount, as arterial thrombosis is a preventable phenomenon with adequate and appropriate thromboprophylaxis (Phillips et al., 2005). Such a need for targeted optimization of post-revascularization thromboprophylaxis is crucial to avoid similarly catastrophic bleeding events when the benefit of escalated antiplatelet therapy is not warranted in low-risk patients (Brown et al., 2018). Specifically in this study, we demonstrated that MLMs derived from patient baseline (clinical, demographic, and intervention- related) and TEG-PM parameters can predict the risk of ATEs following LER of patients with symptomatic PAD.

The TEG-PM parameters provide detailed insights into the coagulation cascade and platelet function that conventional coagulation tests cannot capture. Reaction Time (R), which averaged 7.7 min in non-ATE patients versus 8.7 min in ATE patients (*p* = 0.09), represents the time to initial fibrin formation and reflects the activity of clotting factors. The longer R time in ATE patients suggests that traditional coagulation cascade activation may not be the primary driver of thrombotic events in PAD patients post-revascularization. The CRT MA (63.7 mm vs. 64.8 mm in non-ATE vs. ATE groups) and ADP MA (44.0 mm vs. 42.3 mm) values suggest that overall clot strength and ADP-mediated platelet activation may play important roles in post-revascularization thrombosis. These findings have direct clinical implications: patients with elevated MA values might benefit from more aggressive antiplatelet therapy, while those with lower values might be adequately managed with standard prophylaxis. Lysis at 30 min (LY30) was notably lower in the ATE group (0.5% vs. 0.8%), suggesting that impaired fibrinolysis might contribute to thrombotic risk. This finding could identify patients who might benefit from targeted fibrinolytic therapy or more intensive monitoring.

Several studies have proven that MLMs help identify individuals at high risk, specifically for DVT, Wang et al. (2023) achieved an AUC of 0.92 for knee/ hip arthroplasty, Jin et al. (2022) 0.77 for hospitalized cancer patients, Ryan et al. (2021) 0.83–0.85 for hospitalized hospitals, and Liu et al. (2019) 0.77 for catheter related thrombosis, demonstrating the potential of artificial intelligence technology to improve thromboprophylaxis by having more personalized approach and reduce healthcare burdens (Blaisdell et al., 1978). Interestingly and to the best of our knowledge, no group has attempted to incorporate TEG-PM evaluations with patient data at baseline to train a prediction model for thrombosis, whether arterial or venous. This study illustrated that the LR model showed the best prediction performance compared to the XGBoost and Decision Trees models, especially when traditional baseline variables are combined with TEG-PM parameters collected periodically over the course of follow-up, with an optimized AUC of 0.76, sensitivity of 68%, and specificity of 71%. The LR MLM performed relatively poorly when trained using only baseline characteristics with an AUC of 0.51. On the other hand, when the LR MLM was trained using only the most relevant TEG-PM parameters, it showed better performance with an AUC of 0.68. Utilizing MLMs builds on this inferential approach to TEG-PM values at a higher level, unveiling the hidden predictive value of multiple TEG-PM parameters simultaneously beyond the interpretation of individual parameters and in the context of patient characteristics.

## Limitations

The diagnosis of ATE was only considered when thrombosis complicates the initially revascularized arterial lesion site and subsequent management using fluoroscopic evaluation confirms occlusion of the index arterial lesion site. ATEs occurring in other regions along the arterial tree of the lower extremity were considered separate index events and not ATE complications during follow up. Additionally, our sample size is relatively lower than those used for similar studies developing predictive models. However, this study prospectively recruited consecutively presenting PAD patients and employed the two measures to minimize sample size bias, including random sampling using repeated k-fold cross-validation and SMOTE analysis. Lastly, the limited racial diversity of the study population affects the generalizability of the findings. Future research should focus on refining these predictive models with a larger sample size and a more diverse population to improve clinical outcomes and reduce the burden of ATE.

The statistical power and demographic composition of our study cohort warrant careful consideration. While our sample size of 308 patients with 56 ATE events (18.3%) was sufficient to develop and validate our predictive models, particularly with the use of SMOTE and cross-validation techniques, larger cohorts would be valuable for more robust validation and incorporation of multivariate analysis in the variable selection process. Moreover, our study population was predominantly White (84%), which limits the generalizability of our findings to other racial groups. This is particularly relevant given that PAD prevalence, presentation, and outcomes can vary significantly across different racial and ethnic populations. Future multi-center studies should prioritize the recruitment of diverse patient populations to validate and potentially recalibrate these predictive models across different demographic groups. Additionally, future research could explore alternative models to evaluate their comparative effectiveness in predicting arterial thrombotic events.

## Conclusion

The LR model showed the best prediction performance compared to the XGBoost and Decision Trees models, specifically when including the TEG-PM values. Incorporating TEG-PM values into MLMs could offer a promising approach to identifying high-risk patients, enabling tailored thromboprophylaxis interventions.

## Data Availability

The raw data supporting the conclusions of this article will be made available by the authors, without undue reservation.
