# Supplementary material for: Using machine learning models to predict post-revascularization thrombosis in PAD
Source: Front Artif Intell. 2025 May 7;8:1540503. doi: 10.3389/frai.2025.1540503 (PMC12092403; doi:10.3389/frai.2025.1540503)
Supplement: Supplementary file 1 [file Table_1.docx]

**Supplementary Appendix**

| **Table S1.** A list of laboratory values assessed for all participants at the time of TEG-PM evaluation. | | | | |
| --- | --- | --- | --- | --- |
| Lab Value | No ATE Group (n=252) | ATE Group (n=56) | Total (n=308) | p value |
| HbA1c Baseline | 7.2 (0.5) | 7.1 (0.3) | 7.2 (0.5) | .32 |
| INR value | 1.4 (0.2) | 1.4 (0.4) | 1.4 (0.3) | .95 |
| aPTT | 60.6 (13.9) | 61.4 (19.1) | 60.7 (15.1) | .77 |
| White blood cells in K/uL | 9.3 (2.1) | 9.6 (2.0) | 9.3 (2.1) | .29 |
| Red blood cells (M/uL) | 3.7 (0.3) | 3.7 (0.4) | 3.7 (0.3) | .97 |
| Hemoglobin (g/dL) | 10.5 (1.3) | 10.5 (0.9) | 10.5 (1.2) | .81 |
| Hematocrit % | 33.1 (3.3) | 33.0 (2.7) | 33.1 (3.2) | .89 |
| Platelet (K/uL) | 266.2 (81.4) | 280.4 (59.0) | 269.1 (77.4) | .13 |

Data are presented as mean ± standard deviation (SD). HbA1c= hemoglobin A1c; INR= International normalized ratio; aPTT= activated partial thromboplastin clotting time.
